# The Effect of Neighborhood Deprivation on Mortality in Newly Diagnosed Diabetes Patients: A Countrywide Population-Based Korean Retrospective Cohort Study, 2002–2013

**DOI:** 10.3390/ijerph19074324

**Published:** 2022-04-04

**Authors:** Kyoung-Hee Cho, Juyeong Kim, Young Choi, Tae-Hyun Kim

**Affiliations:** 1 Department of Health Policy and Management, Sangji University, Wonju-si 26339, Korea; chokh017@sangji.ac.kr; 2 Institute of Health Services Research, Yonsei University, Seoul 03722, Korea; kjy394@syu.ac.kr (J.K.); ychoi@cup.ac.kr (Y.C.); 3 Department of Public Health, Sahmyook University, Seoul 01795, Korea; 4 Department of Healthcare Management, Catholic University of Pusan, Busan 46252, Korea; 5 Graduate School of Public Health, Yonsei University, Seoul 03722, Korea

**Keywords:** socioeconomic status, neighborhood deprivation, combined effect, all-cause mortality, diabetes

## Abstract

Background: Neighborhood environmental factors along with individual factors are beginning to make a mark as factors which influence individual health outcomes. The goal of this study is to look at the combined impact of individual and neighborhood socioeconomic status on all-cause mortality in diabetic patients who have just been diagnosed. Methods: The Korean National Health Insurance (2002–2013) was employed in this cohort research, which used a stratified random sample. During the years 2003–2006, a total of 15,882 individuals who were newly diagnosed with diabetes and using oral disease-controlling medication were included in the study. Individual income and neighborhood deprivation index were used to examine the combined effect on all-cause mortality. The frailty model was performed using Cox’s proportional hazard regression. Results: During the study period, 28.3 percent (*n* = 4493) of the 15,882 eligible individuals died. In a Cox regression analysis after adjusting for all covariates, with advantaged and disadvantaged neighborhoods classified according to individual household income, the adjusted HR for patients living in a disadvantaged area was higher compared to patients living in an advantaged area in patients with middle income, compared to the reference group (a high income within an advantaged neighborhood) (HR, 1.22; 95% CI, 1.09–1.35; HR, 1.13; 95% CI, 1.02–1.25, respectively). The adjusted HR for patients with low income who lived in a disadvantaged location was greater than for patients who lived in an advantaged area (HR, 1.34; 95% CI, 1.18–1.53 vs. HR, 1.28; 95% CI, 1.14–1.49). Conclusions: Individual SES has a greater impact on all-cause mortality among diabetic patients when they live in a low-income neighborhood.

## 1. Introduction

Diabetes mellitus is a typical chronic disease, and disease burden due to this disease is considered a major public health challenge in developed countries. According to the statistics on the cause of death, the number of deaths due to diabetes in 2020 was 8456 per 100,000 people, accounting for 2.8% of the total cause of death, and ranked sixth. In the same year, the number of deaths from heart disease reached 32,347 per 100,000 people, and the number of deaths from cerebrovascular disease reached 21,860 per 100,000 people [1]. Diabetes mellitus is a dangerous disease that in itself leads to death, but it is also a disease that requires management as a risk factor that leads to death from cardio-cerebrovascular disease because diabetes acts as a risk factor for the occurrence of cardio-cerebrovascular disease [2,3,4,5]. Several studies have found a socioeconomic gradient in diabetes risk variables. Most previous research examining socioeconomic differences in diabetes incidence or prevalence [6,7,8,9,10,11,12] revealed that those with poor income [6,7,8,9,12], low education [6,8,12], and blue-collar jobs, along with those who live in deprived areas [13], have a higher risk of acquiring diabetes. In the west, attempts to explain the inverse relationship have been popular. Health behaviors [13,14,15], material conditions [15], psychological qualities [16], and early life exposures [17] have all been studied to see how they work. Most low SES was a risk factor for the onset of certain aliments and these diseases could affect mortality in itself as well as various complications due to these diseases affecting mortality.

In the past, the focus has been on individual characteristics as risk factors for health [18,19,20,21]. Recently, many studies have showed that neighborhood factors lead to health disparities [22,23,24,25,26]. Thus, these neighborhood factors cannot be overlooked. In the health determinants model of the Dahlgren and Whitehead model [27], the factors determining health are classified into five areas: (1) innate personal characteristics such as age, sex, race, and genetic factors (2) individual lifestyle factors such as smoking, alcohol consumption, and physical activity (3) social and community networks such as family and wider social circles (4) living and working conditions such as access and opportunities related to jobs, housing, education, and welfare services and (5) general socioeconomic, cultural, and environmental conditions such as disposable income, taxation, and availability of work. In particular, the Committee on Social Health Determinants of the World Health Organization (WHO) found that individual access to health resources differs depending on the gap between countries, regions, and classes of socioeconomic resources such as capital, goods, and services, and this is claimed to cause health inequality [28,29].

In previous studies on health inequality, research used a multilevel analysis methodology [30,31] that could determine the influence of individual and environmental factors affecting health outcomes by separating them and using an ecological methodology [32,33]. However, the ecological studies revealed that there was a limit in terms of not reflecting individual factors on health outcomes. At the regional level, factors affecting health were analyzed as a single factor to reveal their relationship with health outcomes [18,19,20,21,22,23,24,25,26], and there were also studies that reflected these neighborhood factors into a single composite index and revealed their relationship [34,35]. In studies analyzed with a single factor, the relationship between each factor and death could be explained, but there was a limitation that it could not comprehensively explain various factors. While it was possible to explain the relationship between health outcomes, there was a disadvantage that it was unknown which factors specifically affect health outcomes. On the other hand, in Korea, the majority of studies have shown health inequality according to an individual’s socioeconomic status without reflecting the neighborhood factors.

Several recent studies have highlighted the distinct effects of individual and neighborhood-level socioeconomic status (SES) on several aspects of diabetes care, including treatment approach, care quality, and mortality. However, results from research that examined neighborhood-level characteristics and their impact on individual health have been mixed. Many people feel that community competition has an independent impact on the health of all citizens [36,37,38,39]. Others have claimed that neighborhood-level impacts are completely attributable to compositional effects, or the aggregation of individual socioeconomic background and individual health status correlations [40,41]. Although SES of individual-level was the same, health outcomes could differ according to SES of neighborhoods because of uneven access to and quality of basic and secondary healthcare. Research studies on neighborhood deprivation were limited to cancer [42,43,44,45,46], cardiovascular diseases [39,47,48], and rheumatoid arthritis [49,50]. The results were inconsistent. To the best of our knowledge, no study that investigated this topic combined the effect of individual and neighborhood SES on mortality among patients with diabetes.

The first aim of this study was to investigate a possible association between individual-level SES and neighborhood-level SES, and mortality in patients with newly diagnosed diabetes. The second aim of this study was to examine if individual and neighborhood SES have a combined influence on diabetes patient mortality.

## 2. Methods

### 2.1. Data Source

The Korean National Health Insurance (KNHI) claims database for 2002–2013 and the 2005 Korea Census were utilized in this investigation. The National Health Insurance Corporation obtains data from cohorts that are representative of the population of the country. These records contain information on 1,025,340 individuals who were chosen from a stratified random sample based on age, gender, area, health insurance type, income quintiles, and individual total medical expenses in 2002. The database contains reimbursement information for each medical treatment, including basic demographic patient information, a clinic or hospital identity, an illness code, expenses incurred, results of health screenings, past/family health history, health habits, and death information. We studied the relationship between combined individual and neighborhood socioeconomic level and mortality in newly diagnosed diabetes patients in a cohort study. The Institutional Review Board of Yonsei University’s Graduate School of Public Health granted this project ethical approval (IRB approval code: 2-1040939-AB-N-01-2016-161). Because the study was based on routinely available administrative and claims data, informed permission was not required.

### 2.2. Study Sample

**Figure 1:**
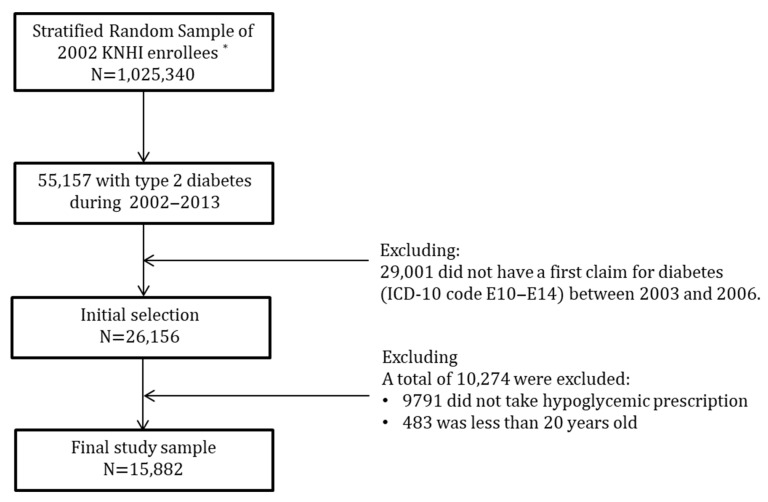
Flowchart for sample selection. *, the Korean National Health Insurance, this is an universal health insurance plan that covers almost all the population and medical facilities.

A total of 55,157 diabetics were included in the KNHI enrollee database. Between 2003 and 2006, 26,156 people with newly diagnosed diabetes (code E10-E14; International Classification of Disease, 10th edition) were chosen. A lack of diabetes claims in 2002–2005, a first diabetes claim in 2003–2006, and the absence of diabetes in the health history prior to the year of diagnosis were all used to confirm new diagnoses. The subjects were followed for a minimum of seven years and a maximum of ten; 10,274 of the 26,156 individuals were eliminated because 483 were under the age of 20 and 9791 patients did not follow their hypoglycemic prescription. These exclusion criteria were required in order to identify true diabetic patients. A total of 15,882 people were included in the final study sample (Figure 1).

### 2.3. Dependent Variable

In this study, the primary outcome was all-cause mortality. The survival time from diagnosis to death, or study end-date, was the outcome variable, and mortality was defined as all-cause mortality as determined from death certificate data in the national death registry. Ischemic heart disease (ICD-10 code I20–I25), cerebrovascular disease (ICD-10 code I60–I69), and diabetes (ICD-10 code E10–E14) were defined as diabetes-related mortality.

### 2.4. Individual Socioeconomic Status

As a proxy variable, the average monthly insurance premium for household income was utilized. In Korea, there are two types of health insurance: National health insurance and medical aid. Medical aid is available to anyone with a household income of less than Korea Won (KRW) 600 per month based on a single family. People with household income of more than KRW 600 per month can apply for basic livelihood security recipient, and if somebody is eligible for basic livelihood security recipient, they are automatically entitled to medical aid [51]. People who have national health insurance via their jobs pay monthly insurance payments depending on their yearly earnings, whereas those who are self-employed pay rates based on their assets. Those who were eligible for national health insurance were placed in the 1 percentile to 100 percentile range, while those who received medical aid were placed in the 0 percentile. Individual family income was divided into three categories (Low, 0–20 percentile; Middle, 21–80 percentile; High, 81–100 percentile). Administrative districts in Korea are composed of Si, Gun, and Gu. Si was classified as metropolitan, Gu as urban, and Gun as rural. In our country, disability grades are divided into 1 to 6, and the lower the number, the more severe disability. In this study, grades 1 and 2 were classified as severe and the others as mild [52].

### 2.5. Neighborhood Deprivation Index

To quantify deprivation at the neighborhood level, a summary measure was utilized. Using census data from 2005, the modified Carstairs index [53] was used to measure local deprivation. In earlier investigations, four factors from census data were used to calculate the Carstairs index: (1) inhabitants in homes headed by unskilled individuals, (2) jobless males, (3) residents who are overcrowded, and (4) people who do not have access to a vehicle. According to Lee’s study [54], we replaced ”residents without a car” with ”residents not owner occupied” because we could not obtain information on ”residents without a car” from census data. The neighborhood deprivation index was calculated at the Si (city), Gun (county), and Gu (borough) levels by combining these four fundamental factors, similar to how the Carstairs index was generated. According to the 2016 demographics of the Ministry of the Interior, the average population of Si, Gun, and Gu was 240,000; 54,000; and 320,000, respectively [55]. All minor places in Korea are divided into three geographical units: Si, Gun, and Gu. The mean and standard deviation of four indicators were used to generate the z-score at the Si, Gun, and Gu levels. We produced a z-score by subtracting the mean from the observed value for each indicator, dividing by a standard deviation, and then adding the four standardized z-scores. The median for neighborhood deprivation index was used to separate disadvantaged and advantaged communities. There are many community factors that affect health, such as healthcare resources, community infrastructure for healthy behaviors, the quality of the physical environment, and the level of public services. Each element has different characteristics for each region, so we needed one composite index that could reflect all these factors. We considered various composite indices related to neighborhood deprivation. The Townsend index, which reflects the level of local deprivation using the variable ”Do you own a house”, did not reflect the situation in Korea well. This is because real estate prices are much higher for people who rent rather than own in the city, despite living in the countryside and owning their own homes.

### 2.6. Covariates

Age (20–49, 50–59, 60–69, or 70 years), sex, residential area (metropolitan, urban, or rural), Charlson comorbidity index (0, 1, 2, or 3) [56], number of risk factors (none, hypertension or dyslipidemia, hypertension and dyslipidemia), disability (normal, mild, severe), and the number of health screenings during the follow-up period were among the covariates (1, 2, 3, or 4). All diagnostic information was gathered from hospital and outpatient billing data at diagnosis year, and only the comorbidity component of the Charlson comorbidity index was generated.

### 2.7. Statistical Analysis

The chi-square test was used to obtain descriptive statistics for all variables, including frequencies and percentages for categorical variables. The Kaplan–Meier product limit technique was used to assess survival probability for all-cause mortality, using log rank tests stratified by socioeconomic level. Survival analysis was performed using a Cox proportional hazard model by frailty model. This frailty model could evaluate whether there is intra-cluster homogeneity of the outcome between individual socioeconomic status and neighborhood deprivation through the integration of random effect [57]. When this random influence is significant, it increases the region’s vulnerability to short survival time, and when it is modest, it decreases this susceptibility. We adopted the frailty model because the variance and *p*-value for mortality among areas were 0.022 and 0.004, respectively. Scaled Schoenfeld residuals were used to evaluate the proportional hazards assumptions, and no violations were discovered. SAS 9.3 software was used for all statistical analyses.

## 3. Results

During the research period, 4493 (28.3%) of the 15,882 eligible individuals died; 11,389 (71.7%) survived (Table 1). Between the two groups, there were significant differences in all individual patient variables (age, sex, health insurance type, income, Charlson comorbidity index, residential region, number of risk factors, disability, and number of health screenings over the subsequent research period). In the variable combined with the individual level of family income and the degree of neighborhood deprivation, the distribution of the surviving and the dead was different: 15.1% (*n* = 1714) vs. 14.2%(*n* = 637) for those with high household income and lived in an advantaged neighborhood, 17.8% (*n* = 2031) vs. 17.9% (*n* = 803) for those with high household income and lived in a disadvantaged neighborhood, 22.2% (*n* = 2533) vs. 20.1% (*n* = 901) for those with middle household income and lived in an advantaged neighborhood, 29.8% (*n* = 3396) vs. 28.5% (*n* = 1281) for those with middle household income and lived in a disadvantaged neighborhood, 6.1% (*n* = 694) vs. 8.2% (*n* = 370) for those with low household income and lived in an advantaged neighborhood, and 9.0% (*n* = 1021) vs. 11.2% (501) for those who with low household income and lived in a disadvantaged neighborhood.

**Figure 2:**
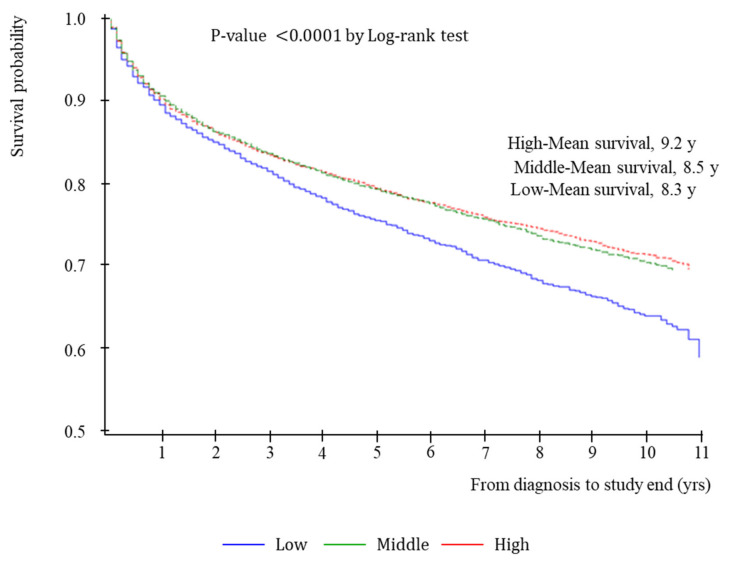
Individual household income stratified survival probability for all-cause mortality.

Individuals with high income, middle income, and low income had 9.2, 8.5, and 8.3 mean years of survival, respectively (*p*-value 0.0001 by log-rank test; Figure 2).

**Figure 3:**
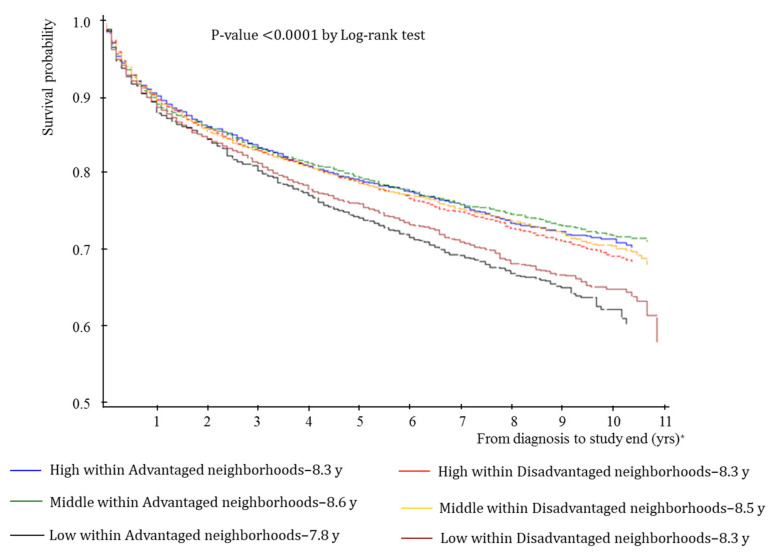
All-cause mortality survival probability stratified by individual household income in advantaged and disadvantaged communities. * yrs: years.

Individuals with low income in a disadvantaged location had an average of 8.3 mean years of survival, whereas those with low income in an advantaged area had an average of 7.8 mean years of survival (*p*-value 0.0001 by log-rank test; Figure 3).

After adjusting for all factors, Table 2 displays the Cox regression analysis, which did not mix individual and neighborhood SES. The adjusted HR for low and middle income was 1.31 (95% CI, 1.20–1.43) and 1.16 (95% CI, 1.09–1.25), respectively, when compared to the reference group with high income. The difference between disadvantaged and advantaged communities, on the other hand, was not statistically significant. The adjusted HR for disadvantaged neighborhood was 1.01 (95% CI, 0.94–1.09) comparing to an advantaged neighborhood. The adjusted HR of all-cause mortality was the highest for those over 70 years of age compared to those between 20~49 years old (aHR 9.86; 95% CI, 8.58–11.32), was higher in men than women (aHR 1.60; 95% CI, 1.50–1.70), and higher in those who lived in rural areas than in those who lived in the metropolitan area (aHR 1.28; 95% CI, 1.16–1.43). In CCI, the higher the CCI score, the higher the mortality hazard ratio (aHR 1.34; 95% CI, 1.23–1.46 vs. aHR 1.71; 95% CI, 1.56–87 vs. aHR 2.78; 95% CI, 2.58–3.00, respectively).

Table 3 shows the HRs of individual family income for all-cause mortality in disadvantaged and affluent areas. When patients with middle income were compared to patients with high income inside an advantaged area, the risk of all-cause mortality for patients living in a disadvantaged neighborhood was higher than for those living in an advantaged neighborhood (HR, 1.22; 95% CI, 1.09–1.35 vs. HR, 1.13, 95% CI, 1.02–1.25, respectively). Furthermore, consistent results were achieved among low-income patients, with the adjusted HR for patients living in a disadvantaged region being greater than the adjusted HR for patients residing in an advantaged location (HR, 1.34; 95% CI, 1.18–1.53 vs. HR, 1.31; 95% CI, 1.14–1.49, respectively). Among the case of diabetes-related mortality, however, statistical significance was found only in low-income patients, and the adjusted HR for patients living in a privileged location was greater than that of patients living in a disadvantaged area (HR, 1.56; 95% CI, 1.07–2.29 vs. HR, 1.51; 95% CI, 1.06–2.15, respectively).

## 4. Discussion

The significance of individual and neighborhood socioeconomic variables in patients with diabetes was investigated in this study, which used a comparative and longitudinal methodology. Patients with diabetes who lived in a poor neighborhood had a greater risk of all-cause death than those who lived in an advantaged location, even if they had the same amount of individual income. Additionally, in patients dwelling in the same disadvantaged area or advantaged area, the lower earning individuals had increased risk of all-cause mortality, even after controlling for individual and neighborhood characteristics. In diabetes-related mortality, the risk was high only in individuals with low-income and living within an advantaged area compared to those having high-income and living within a disadvantaged area.

In previous studies, diabetes was more prevalent in low socioeconomic groups [58,59]. However, the risk of mortality was different in people with equal risk such as diabetes, according to individual income and neighborhood deprivation. Why are the impoverished in rich places more likely to die? Why do various levels of household income appear to have varied contextual impacts on mortality? Because the causal linkages between individual and neighborhood socioeconomic disparity and poor health outcomes are unknown, our findings might point to a variety of causes. The first possibility was the inequality of resources related to medical services [36,60,61]. According to research, poor and wealthy people living in costly areas may have varied access to resources. The majority of social services aimed at assisting the poor are concentrated in underserved regions. People who do not have access to a convenient mode of transportation may not be able to use such services unless they are nearby. However, in Korea, because the country is small and has better mobility between areas, health insurance based on universal coverage that covers the whole nation has increased access to medical treatment. Geographic accessibility has also improved. Although medical treatment is more accessible, there is still a disparity in the number of physicians and medical facilities among communities.

The second argument is that more direct psychological routes resulting from inequality, such as despondency, lack of control, or loss of esteem, have an impact on individual health [62,63,64,65]. The health of the poor in affluent places may be harmed by a lack of social cohesiveness or engagement, which may be connected to psychological disorders. In addition, in disadvantaged communities, psychological stress may be caused by trash and dangerous settings, vandalism, isolation/alienation, and violent crime. In our study, unlike all-cause mortality, people with low income living in disadvantaged areas had a higher risk of mortality than those with low income living in advantaged areas for diabetes-mortality. This result is different from general expectations, which seems to be explainable with a sense of relative deprivation [66]. Even if the income level is the same, depending on the environment in which people live, people living in affluent areas are more likely to feel that their income is worse than the standard of the surrounding environment. This relative deprivation may have led to psychological stress, and increased the risk of death.

The third point, perspectives on the role of socioeconomic position, suggests reasons why the wealthy in more affluent areas may be healthier. Residing in affluent areas would bolster the capacity of the comparatively rich to exploit their expertise, money, power, status, and social connections [67]. The wealthy are more likely to embrace preventive care and take advantage of therapeutic advancements [68], and such information may be easily shared and developed among a network in affluent communities [69]. Individuals with low SES, in contrast, may find it more difficult to receive meaningful ideas or advice from relatives, friends, or acquaintances due to their higher isolation.

The fourth explanation is that a lack of a safe environment limits exercise opportunities, making it more difficult to maintain a healthy lifestyle [42]. Furthermore, sociocultural norms surrounding a healthy lifestyle may differ between communities, affecting people’s health and mortality risk. Environmental risk variables such as community income, neighborhood education, and neighborhood unemployment, for example, have been researched and linked to mortality.

In addition to these four possible explanations, when examining other perspectives related to mortality with diabetes and calculating the CCI score, hypertension and dyslipidemia were not considered and treated as separate risk factors. This is because diabetic patients with high blood pressure or dyslipidemia can develop stroke and cardiovascular diseases, and act as a mediating factor leading to death from these conditions. Similarly, CCI reflected the presence of cancer and the other chronic diseases, which may have contributed to the death.

In the case of Korea, the proportion of government or compulsory insurance funds in current healthcare was 61% in 2019, lower than the average of 74.1% in OECD countries. This means that the burden of medical expenses on households is greater than that of OECD countries. Therefore, there is a need for a policy that increases access to healthcare by reducing the burden on individual low economic levels by strengthening insurance coverage for medical expenses. In addition, at the regional level, health promotion education and related projects are needed to maintain and improve a healthy life, and at the same time, it is considered necessary to expand infrastructure such as sports facilities and good healthcare facilities.

Our research has significant limitations. To begin, we only included high-risk diabetic populations. Our findings cannot be applied to the general population in the absence of diabetes. Second, because this study employed data from a claims database, lifestyle and educational characteristics that impact mortality could not be taken into account. Third, we did not consider modifying the research participants’ neighborhood deprivation status if they relocated inside the study region. Furthermore, although geographic accessibility, transportation to healthcare resources, extreme temperature and natural environment could have a major impact on mortality, we did not consider these variables. Thus, there may be a problem with unmeasured confounding bias. Additionally, we did not reflect the changes over time for CCI or the other risk factors affecting mortality, which may be a limitation of our study. Finally, we used the modified Carstairs index that was used in previous studies, although we did not confirm the reliability and validity of this index. Finally, multicollinearity between health insurance type and household income exists in this analysis because participants in the medical aid group are definitely in the low household income group. This may induce an inconsistent direction of HR between the crude HR and the adjusted HR of medical aid.

Despite its problems, our research offers some advantages. To our knowledge, this research was the first investigation of the link between individual and neighborhood SES and mortality, and it was a prospective design with a comparatively large sample size, resulting in strong statistical power for detecting the impacts of neighborhood deprivation. Second, utilizing nationwide representative cohort data, a representative sample of diabetic patients was studied. Third, we tried to make our research sample more homogeneous by enrolling individuals who had just been diagnosed with diabetes.

## 5. Conclusions

Deprivation in one’s neighborhood leads to all-cause death. Individual and community level variables accumulate weight against people, putting those with both individual- and neighborhood-level risk factors at the highest risk. These findings bring severe clinical and public-health problems, indicating that in the creation of healthcare policy, both individual and neighborhood-level approaches are critical.

## Figures and Tables

**Table 1:** Baseline characteristics of newly diagnosed diabetes.

	Total	Alive		Dead		*p*-Value
Characteristics	*n* = 15,882	*n* = 11,389	(71.7)	*n* = 4493	(28.3)	
**Age, *n* (%)**						
20~49	2932	2697	(23.7)	235	(5.2)	<0.0001
50~59	3786	3262	(28.6)	524	(11.7)	
60~69	4928	3593	(31.6)	1335	(29.7)	
≥70	4236	1837	(16.1)	2399	(53.4)	
**Sex, *n* (%)**						
Male	8102	5604	(49.2)	2498	(55.6)	<0.0001
Female	7780	5785	(50.8)	1995	(44.4)	
**Health insurance type, *n* (%)**						
National health insurance	15,505	11,174	(98.1)	4331	(96.4)	<0.0001
Medical aid	377	215	(1.9)	162	(3.6)	
**Household income, *n* (%)**						
Low (≤20 percentile)	2586	1715	(15.1)	871	(19.4)	<0.0001
Middle (21–80 percentile)	8111	5929	(52.1)	2182	(48.6)	
High (≥81 percentile)	5185	3745	(32.9)	1440	(32.1)	
**Carstairs index, *n* (%)**						
Disadvantaged neighborhood (below mean)	9033	6448	(56.6)	2585	(57.5)	0.293
Advantaged neighborhood (above mean)	6849	4941	(43.4)	1908	(42.5)	
**Combined individual household income level–neighborhood deprivation, *n* (%)**						
High–Advantaged neighborhood	2351	1714	(15.1)	637	(14.2)	<0.0001
High–Disadvantaged neighborhood	2834	2031	(17.8)	803	(17.9)	
Middle–Advantaged neighborhood	3434	2533	(22.2)	901	(20.1)	
Middle–Disadvantaged neighborhood	4677	3396	(29.8)	1281	(28.5)	
Low–Advantaged neighborhood	1064	694	(6.1)	370	(8.2)	
Low–Disadvantaged neighborhood	1522	1021	(9.0)	501	(11.2)	
**Residential area, *n* (%)**						
Metropolitan	7265	5339	(46.9)	1926	(42.9)	<0.0001
Urban	6642	4750	(41.7)	1892	(42.1)	
Rural	1975	1300	(11.4)	675	(15.0)	
**Charlson comorbidity index ^†^, *n* (%)**						
0–1	8622	6931	(60.9)	1691	(37.6)	<0.0001
2	3197	2354	(20.7)	843	(18.8)	
3	1805	1175	(10.3)	630	(14.0)	
≥4	2258	929	(8.2)	1329	(29.6)	
**Number of risk factors, *n* (%)**						
none	2574	1333	(11.7)	1241	(27.6)	<0.0001
with hypertension or dyslipidemia	9834	6994	(61.4)	2840	(63.2)	
with hypertension and dyslipidemia	3474	3062	(26.9)	412	(9.2)	
**Disability, *n* (%)**						
Normal	14,127	10,466	(91.9)	3661	(81.5)	<0.0001
Mild disability	1150	701	(6.2)	449	(10)	
Severe disability	605	222	(2)	383	(8.5)	
**Number of health screening during follow-up** **period, *n* (%)**						
1	9125	5293	(46.5)	3832	(85.3)	<0.0001
2	2286	1925	(16.9)	361	(8.0)	
≥3	4471	4171	(36.6)	300	(6.7)	

^†^, calculated by extracted diabetes, hypertension, and hyperlipidemia among comorbidity components.

**Table 2:** All-cause mortality among newly diagnosed diabetics: hazard ratio (*n* = 15,882).

	Unadjusted		Adjusted	
Characteristics	HR	95% CI	HR	95% CI
**Age**				
20~49	1.00		1.00	
50~59	1.79	(1.53–2.09)	2.44	(2.09–2.86)
60~69	3.79	(3.30–4.36)	4.59	(3.98–5.29)
≥70	9.89	(8.65–11.31)	9.86	(8.58–11.32)
**Sex**				
Male	1.26	(1.19–1.34)	1.60	(1.50–1.70)
Female	1.00		1.00	
**Health insurance type**				
National health insurance	1.00		1.00	
Medical aid	1.54	(1.32–1.80)	0.81	(0.68–0.97)
**Household income**				
≤20 percentile	1.25	(1.15–1.36)	1.31	(1.20–1.43)
21–80 percentile	0.97	(0.91–1.04)	1.16	(1.09–1.25)
≥81 percentile	1.00		1.00	
**Carstairs index**				
Disadvantaged neighborhood (below mean)	0.99	(0.91–1.07)	1.01	(0.94–1.09)
Advantaged neighborhood (above mean)	1.00			
**Residential area**				
Metropolitan	1.00		1.00	
Urban	1.09	(1.03–1.17)	1.06	(0.98–1.15)
Rural	1.37	(1.25–1.50)	1.28	(1.16–1.43)
**Charlson comorbidity index ^†^**				
0–1	1.00		1.00	
2	1.41	(1.30–1.53)	1.34	(1.23–1.46)
3	1.99	(1.81–2.18)	1.71	(1.56–1.87)
≥4	4.34	(4.04–4.67)	2.78	(2.58–3.00)
**Number of possessed risk factors**				
none	1.00		1.00	
those who have hypertension or dyslipidemia	0.46	(0.43–0.49)	0.42	(0.39–0.45)
those who have hypertension and dyslipidemia	0.17	(0.15–0.19)	0.21	(0.19–0.24)
**Disability**				
Normal	1.00		1.00	
Mild	1.68	(1.52–1.85)	1.30	(1.18–1.44)
Severe	3.17	(2.85–3.52)	1.65	(1.48–1.83)
**Health screening during follow-up period**				
1	1.00		1.00	
2	0.31	(0.28–0.34)	0.37	(0.34–0.42)
≥3	0.12	(0.11–0.14)	0.17	(0.15–0.19)

^†^, comorbidity components such as diabetes, hypertension, and hyperlipidemia were extracted and used to generate the score.

**Table 3:** Mortality HRs adjusted for individual household income in disadvantaged and affluent communities.

	Disadvantaged Neighborhoods ^†^				Advantaged Neighborhoods ^†^			
All-Cause Mortality	No. of Deaths (Deaths per 1000 py *)		Adjusted HR **	95% CI	No. of Deaths (Deaths per 1000 py *)		Adjusted HR **	95% CI
Individual household income								
High (≥81 percentile)	803	(39.6)	1.02	(0.91–1.15)	637	(37.7)	1.00	
Middle (21–80 percentile)	1281	(38.5)	1.22	(1.09–1.35)	901	(36.5)	1.13	(1.02–1.25)
Low (≤20 percentile)	501	(47.6)	1.34	(1.18–1.53)	370	(50.9)	1.31	(1.14–1.49)
**Diabetes-related mortality ^‡^**								
Individual household income								
High (≥81 percentile)	91	(4.5)	1.05	(0.76–1.45)	69	(4.1)	1.00	
Middle (21–80 percentile)	123	(3.7)	1.02	(0.75–1.38)	106	(4.3)	1.17	(0.86–1.59)
Low (≤20 percentile)	59	(5.6)	1.51	(1.06–2.15)	45	(6.2)	1.56	(1.07–2.29)

*, person years; **, age, sex, health insurance type, residential location, Charlson comorbidity index, number of risk factors, disability, and number of health screenings throughout the follow-up period were all controlled; ^†^, the mean for neighborhood Carstairs index was used to separate disadvantaged and advantaged neighborhoods, with disadvantaged areas having a higher Carstairs index than the mean—a higher Carstairs index indicates a more impoverished community; ^‡^, deaths caused by cerebrovascular disorders, death caused by cardiovascular diseases, and death caused by diabetes are all included in diabetes-related mortality.

## Data Availability

We used the data that the National Health Insurance Service provided for researchers. NHIS give the researchers a certain period to use data. Currently, this data could not be used since the validity period of the data have passed.
